# Advanced MR imaging features of uncontrolled phenylketonuria in an adult patient

**DOI:** 10.1055/s-0043-1761293

**Published:** 2023-03-14

**Authors:** Alper Ayasli, Hayri Ogul, Omer Onbas

**Affiliations:** 1 Duzce University, Medical Faculty, Department of Neurology, Duzce, Turkey.; 2 Duzce University, Medical Faculty, Department of Radiology, Duzce, Turkey.

**Figure 1:**
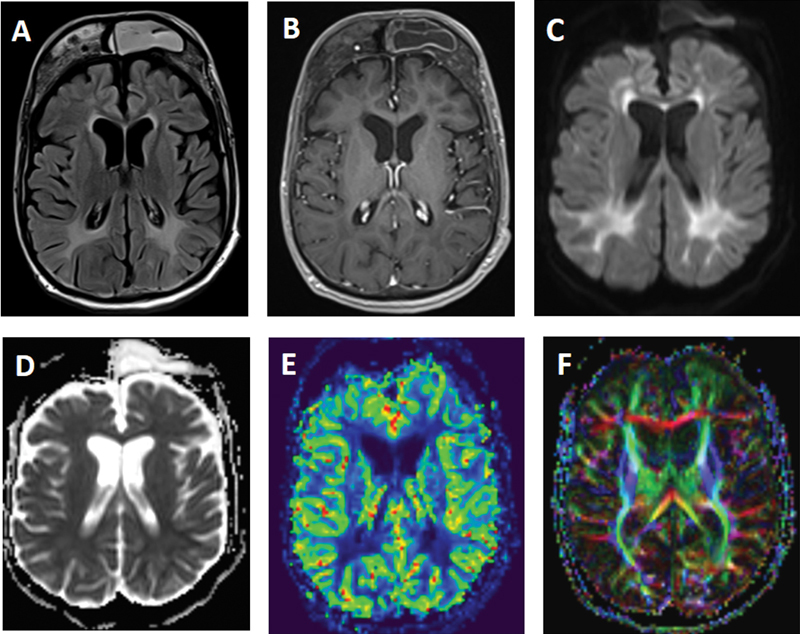
Axial fluid attenuation inversion recovery (A) MR image shows extensively increased periventricular white matter signal abnormalities in both cerebral hemispheres. No contrast-enhancing lesion is observed in post-contrast T1-weighted (B) MR imaging. Axial fluid attenuation inversion recovery and post-contrast T1-weighted MR images also demonstrate a prominent hyperostosis of the frontal bone. Diffusion weight imaging (C) shows bright signals in the periventricular white matter representing restricted diffusion with slightly hypointense ADC map (D). rCBF perfusion (E) MR reveals slightly hypoperfused white matter areas. Diffusion tensor imaging (F) shows thinning white matter fibers.

A 29-year-old male was brought to the neurology department with a complaint of vomiting for one month. He had known mental retardation, epilepsy, and chronic hypocalcemia. His relatives said that the patient was diagnosed with phenylketonuria in childhood. As a result of further radiological examination, cranial MR images were compatible with phenylketonuria ( Figure 1A-F ). Although our case is very rare, it is worth reminding that phenylketonuria can be diagnosed in adulthood 1 and to emphasize that phenylketonuria is also present in the differential diagnosis of leukoencephalopathy.
